# The politics of local hospital reform: a case study of hospital reorganization following the 2002 Norwegian hospital reform

**DOI:** 10.1186/1472-6963-9-212

**Published:** 2009-11-20

**Authors:** Trond Tjerbo

**Affiliations:** 1 Institute of Health Management and Health Economics, University of Oslo, Oslo, Norway; 2 Norwegian Institute for Urban and Regional Affairs, Oslo, Norway

## Abstract

**Background:**

The Norwegian hospital reform of 2002 was an attempt to make restructuring of hospitals easier by removing politicians from the decision-making processes. To facilitate changes seen as necessary but politically difficult, the central state took over ownership of the hospitals and stripped the county politicians of what had been their main responsibility for decades. This meant that decisions regarding hospital structure and organization were now being taken by professional administrators and not by politically elected representatives. The question raised here is whether this has had any effect on the speed of restructuring of the hospital sector.

**Method:**

The empirical part is a case study of the restructuring process in Innlandet Hospital Trust (IHT), which was one of the largest enterprise established after the hospital reform and where the vision for restructuring was clearly set. Different sources of qualitative data are used in the analysis. These include interviews with key actors, observational data and document studies.

**Results:**

The analysis demonstrates how the new professional leaders at first acted in accordance with the intentions of the hospital reform, but soon chose to avoid the more ambitious plans for restructuring the hospital structure and in fact reintroduced local politics into the decision-making process. The analysis further illustrates how local networks and engagement of political representatives from all levels of government complicated the decision-making process surrounding local structural reforms. Local political representatives teamed up with other actors and created powerful networks. At the same time, national politicians had incentives to involve themselves in the processes as supporters of the status quo.

**Conclusion:**

Because of the incentives that faced political actors and the controversial nature of major hospital reforms, the removal of local politicians and the centralization of ownership did not necessarily facilitate reforms in the hospital structure. Keeping politics at an arm's length may simply be unrealistic and further complicate the politics of local hospital reforms.

## Background

Because of a decentralized settlement pattern and great geographical distances, local hospitals have played an important role in the provision of specialist health care in Norway. However, the decentralized hospital structure may have negative effects on clinical quality [1,2], and it is generally regarded as costly. The need for structural changes within the hospital sector was emphasized in several reviews and royal commissions leading up to the 2002 hospital reform, when the central state took over ownership of the hospitals [3-6]. As underlined in the report from the royal commission that considered the central government takeover of hospitals [4], there was a decision-making problem at the county level because politicians were required to balance the national goals of an efficient hospital structure against local political factors. The effect was "...decisions that lead to low efficiency due to unnecessary duplication of services and too high differentiation of the emergency capacity" [4].

The need for change in the organization and division of labor between hospitals was one of the main reasons for the 2002 reform, although it was not (probably for political reasons) explicitly stated in the act transferring ownership [7]. However, the reform not only transferred ownership from 19 counties to the central state. Two other elements in the reform were of equal importance. First, hospitals were set up as health enterprises or trusts and organized within five Regional Health Authorities (RHA) (enterprises also). Second, both the health enterprises and the RHAs were to be governed by boards comprising professional members. The Minister of Health, acting as their general assemblies^i^, appointed the board members at the RHA level. This implied that politicians were no longer represented at the regional and local level in the health care organization.

The need for restructuring did become explicit, as the RHAs were to organize their service-producing units or health enterprises. As demonstrated by Nerland [8], the goals of higher cost efficiency and higher clinical quality guided the organizational structure for the health enterprises chosen by the RHA. All RHAs concluded that these goals were best accomplished by organizing hospitals within large, geographically organized health enterprises that included, on average, three or four acute hospitals in addition to psychiatric hospitals and other specialist care institutions. A reduction in the duplication of services and increased scale effects were the main arguments for this choice of organizational structure [8]. Both the reduction of local political involvement and the move from a (formal) multileveled governance structure to a single level structure could make restructuring easier. That is, removing local politicians from structural decisions could also remove them as opponents of restructuring. By introducing one formal decision maker, the central state, it was assumed that gaming between the governmental levels would be also reduced.

The question asked in this paper is whether the new organizational model has worked as intended. The 2002 Norwegian hospital reform involved removing local politics from the decision-making process by transferring ownership of the hospitals to the state, organizing hospitals as enterprises, and introducing professional board members. Did this facilitate restructuring of the hospital sector? Alternatively, did restructuring of the hospital structure remain as difficult as it was before the central state took ownership?

The paper commences with a description of the formal organizational changes that took place as the central state took over ownership of the hospitals in 2002 and a description of general trends in the restructuring of hospitals. A theoretical discussion of how the new organizational model may work follows. The empirical part is a case study of the restructuring process in Innlandet Hospital Trust (IHT), which was the largest enterprise established after the hospital reform and where the vision for restructuring was clearly set. In the discussion, I question whether the "national goal of an efficient hospital structure", as stated in the commission report that considered the central government takeover of the hospital sector [4], really exists.

### The Norwegian hospital reform

The primary goals for Norwegian health and hospital policy over the past 50-60 years has been quality, cost efficiency and equal access for all citizens regardless of personal resources and place of residence. The means that have been put to use to achieve these goals has however varied somewhat more in the same period [8]. In the last part of the nineties the need for organizational reforms and improved cost control became increasingly important, and a change in ownership and the division of tasks between the different levels of government was seen by many as a potential instrument to achieve these goals. The hospital reform of 2002 was planned, passed and implemented at a remarkable pace after the Norwegian Labour party came into power^ii ^[9,10].

In 2002, five health regions, which had existed since the mid 1970s as network organizations between counties, were upgraded to Regional Health Authorities (RHA). The RHA were organized as enterprises with the responsibility of providing hospital care to the inhabitants in their catchments areas, either through hospitals under their own ownership and control or through contracts with private providers [7]. Importantly, as county ownership of the hospitals was removed, local (county) politicians were also removed. The Minister of Health became the general assembly of the RHA, each with a board composed of professional members from business, academia, and the hospital labor organizations. Decisions concerning administrative, organizational, and production issues were decentralized to the RHAs, or even further to the local hospital trusts, which were organized similarly to the RHAs and governed by boards composed of professional members. The political responsibility for the sector was now placed at a single level of government but, at the same time, the degree of delegation to nonpolitical actors was high. In sum the reform therefore also constituted an attempt to keep politics at arm's length.

By concentrating responsibility for financing and production at the central state level, the reformers wanted to improve the overall governing of the sector. The division of the responsibility for production and financing between the counties and the central state was regarded as an obstacle for good governance in the sector. There were also other stated goals behind the reform. For instance, in the act that switched ownership, it is stated that the cabinet's ambition is to "combine availability, quality and care for the patient with a rational resource allocation and a holistic societal responsibility and leadership" [7]. It is clear, however, that the main goal behind the reform was to improve the overall governance of the sector and reduce problems that arose because of the preexisting multileveled governing structure within the sector. But the hospital reform did not deliver as far as the budgetary discipline in the sector is concerned. As it can be seen from figure 2^iii ^[11], the deficits in the sector persisted also after the central state took over ownership

**Figure 1:**
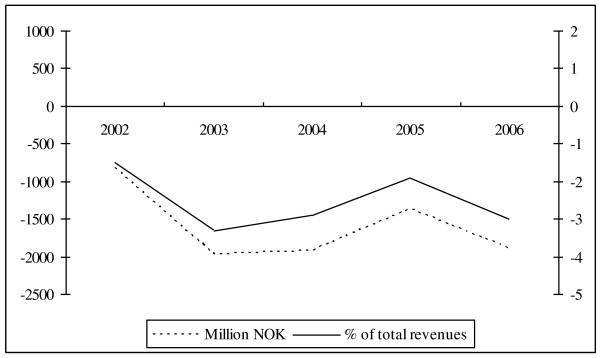
**Running deficits (current prices) in NOK million (left axis) and percent of total revenues (right axis), 2002-2006**.

Now, six years after the reform, hospital debts are higher than ever and the speed of restructuring can hardly be said to be gathering pace. For example, Huseby and Jensberg [12] analyzed the effects of the hospital reform on the degree of centralization of specialized procedures. They concluded there had been no significant change in the level of service concentration and that the overall changes in this area between 1999 and 2005 were low. Another illustrative case is the number of birth units in the Norwegian health care sector. Birth departments are especially relevant in this context for two reasons. First, these services are costly, and increasing efficiency in this area can therefore create significant savings for hospitals. Second, they are one of the most difficult services to centralize because proximity and traveling distance are important aspects of the perceived quality of these services. Further, this is a service that most if not all voters know they themselves (or someone in their family) will probably need in the future. Local reductions and/or restructuring of these services have also proven to be politically difficult in the past. Thus, these cases represent something of a "critical test" for hospital reform. If the new ownership structure facilitates restructuring, then this is where it will be put to the test.

**Figure 2:**
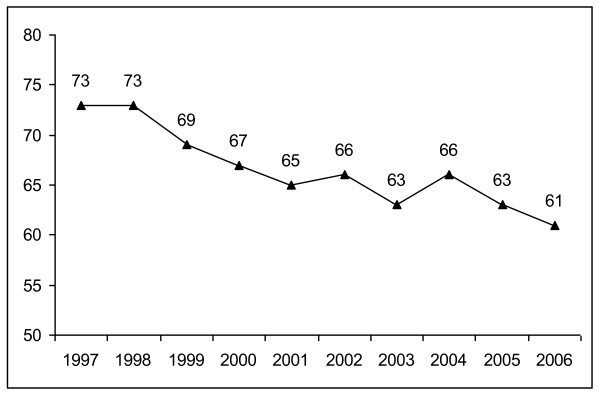
**Number of birth departments in Norway, 1997-2006**. Source: The Medical Birth Register.

Figure 2 shows the number of birth departments in Norway between 1997 and 2006. As indicated, there has been no dramatic difference between the years prior to the central state takeover and the first few years after the takeover. Indeed, the overall trend is a reduction in the number of birth units, and this is similar before and after 2002.

Clearly, the change in ownership has not eradicated the deficits of the sector, nor does it appear that the degree of local changes in the hospital sector have been as widespread and as significant as the premises underlying the reform would lead one to expect. The deficits in the sector did however spur several local reform initiatives. In the next sections I will present some of the insights that can be derived from one of the most ambitious reform initiatives in the hospital sector. First however, I lay out some theoretical considerations about why restructuring in the hospital sector can be politically extremely difficult.

### The politics of restructuring hospitals

The politics of restructuring hospital services have much in common with "pork barrel" politics, which describes the political process of "giving" specific projects to specific areas in return for electoral rewards [13,14]. Politicians who succeed in such a game are often rewarded by (re)election. However, in the restructuring of hospitals, the question is not only about the geographic locality rewarded with investment, but also about the sites that are closed. Such issues are extremely controversial locally. The demands placed on their elected politicians from local voters to retain the status quo are high and the politicians have a strong incentive to engage in the processes. Although a few "unpopular" changes in hospital structure took place during the period of county ownership [15], the problem spelled out and referred to in the introduction was that restructuring was sluggish.

### From multilevel to single level governance

As the central state took over ownership of the hospitals, local political actors no longer played a formal role in decisions on the restructuring of hospital services. This could facilitate hospital restructuring as described earlier. However, the central state is also governed by politicians and there is no a priori reason to believe that their motives differ considerably from county politicians. As discussed in Strøm [16], politicians' motives can be pinned to policy, positions, and votes (reelection), with reelection being particularly important [17]. In health politics, this has the potential to block unpopular reforms. An interesting description of this in the British healthcare system is given by Enthoven [18].

MPs and ministers cheerfully use the NHS as a bundle of gifts to give voters: a new hospital here, stopping those dreadful bureaucrats from closing one down there, winter money here and reductions in waiting lists there. All this is well and democratic, but it isn't the same as a relentless and systematic effort to maximize health gain for money or systematically improving customer service, and indeed may be the opposite.

Two mechanisms in the new hospital organization were intended to hinder political intervention in the decision-making process. First, through the organization of five RHAs with the Minister of Health as the general assembly, politicians were to govern "at a distance" and concentrate on strategic management. Second, the introduction of boards composed of professional members involved the introduction of actors that did not need to fear electoral retaliation if their policies and decisions were not in accordance with the voters' preferences. Consequently, the trade-off between the national goals of an efficient hospital structure and local demand for a decentralized hospital structure, earlier described as a difficult decision-making problem at the county level [4], could now be resolved. This rests, however, on two important premises: first, that the governing process follows the formal hierarchical structure of the sector, and second, that removing politicians formally from the decision-making process reduces their involvement.

### Local protesters and vote-seeking politicians

However, power and influence over the outcomes of the decision-making process may be more complex than a formal focus on roles and authority would suggest. Other arenas exist, power does not reduce to formal authority alone, and a wide array of actors both within and outside the political sphere interact through policy. This is the key description given in the multilevel governance literature [19,20]. The growing interdependence of governments at different levels as well as between governments and nongovernmental actors creates complex policy processes. This refers to the vertical interaction between different levels of government and to the horizontal interaction between governmental actors and actors outside the governmental system.

The relationship between the central state, subnational governments, and nonelected agencies is therefore far more complex than presumed by a formal organizational structure. Local authorities are working alongside other agencies to the extent that one can argue for a change from "local government" to "local governance" [21-23]. Thus, we can expect that actors and networks outside the hospitals and the RHA will attempt to influence their decisions. According to Clark [24], local parties can serve a function outside the formal political arena by providing what can be described as "problem-solving networks" [24,25]. The local party branches can have an ombudsman function and assist local voters in voicing their preferences. A result can also be that close ties to local interest groups are established [24,26]. To see how this works, we need to consider the preferences of the political actors.

Assuming that politicians at all levels of government have votes and reelection as their primary goals [17,27], they are strongly motivated to intervene in unpopular attempts at local reform. Unpopular changes in the hospital structure are highly visible,^iv ^and create strong local demand for influence over the policy process. Local voters have no reason to support cutbacks in their local area as the potential benefit of cutbacks (a less costly hospital structure) is distributed at the national level, while the real or perceived costs (in the form of reduced services) are local. Blocking unpopular reform attempts is therefore valuable to political actors because there are few local opponents to this resistance, and local votes are important for both renomination and reelection. Thus, politicians at all levels have an incentive to accommodate the wishes of local protest groups. This provides incentives for local and county politicians to join protest groups and networks, and for politicians at the national level to prevent^v ^unpopular local reforms.

Consequently, it is difficult to see if anyone actually benefits from standing firm against local protests. In the long run, the premise behind the stated need of reforms is, of course, that everyone will benefit from increasing the overall efficiency of the sector as well as possibly increasing the quality of the care in some instances. In the short run, however, users/citizens observe reductions in proximity (and therefore increased traveling time) as important indicators of quality. The historic surplus of the national budget also means that any perceived requirement for cost cuts is not clear. Consequently, voters place less value on the possible long-term benefits from reforming and centralizing the hospital sector than on any clear and tangible short term costs. This also means that the possible political benefits for a politician by standing firm against protests in order to create balanced budgets is far lower than the benefits from complying with the protesters' demands. Furthermore, whereas the instructions sent to the RHA during the budgetary process in relation to the demand for budgetary balance and cost cuts are the results of plenary processes, local protests are more likely to engage members of parliament (MP) and other politicians from the area in question and not others. In any case, MPs from other areas face few incentives to safeguard the long-term perspective against the short-term demands of local protesters and politicians.

## Methods

Several different types of data were gathered and are put to use in this article. I followed the development in the trust in the period that is analyzed here. This included observing a meeting between the leadership of the trust and local politicians, a group interview with the leadership of the trust and an interview with one of the primary actors in the local protest movement. Furthermore both the Minister of Health and an MP from the opposition were asked about the reform process in the innland area in interviews.

Various documents are also used as sources. These include local (and national) newspapers, transcripts from meetings in Parliament and meetings in the trust and transcripts from meetings in the "society panel". The methodological strategy which is put to use in the analysis is based on triangulation [28], where the basic idea is to strengthen the validity of the conclusions that are drawn by using multiple sources of evidence.

The data gathered and the analysis techniques used are a result of the object that is being studied and the dependent variable. Understanding the politics of a local reform process such as this requires data that can give sufficient depth and detail, and this is best achieved through the use of qualitative data. Clearly, some quantitative material can provide important background information, but on their own they cannot furnish the basis for gathering sufficient information about the dependent variable. Case studies and qualitative material in general, do however make empirical generalization highly problematic^vi ^[29-33]. Unlike extensive studies with a large N it is not possible to specify the degree of uncertainty that is present when generalizing the findings. Furthermore, while the conclusions drawn from statistical analysis of datasets can easily be repeated by other scientists, this is not always possible in studies that rely on qualitative data material. As pointed out by Stake [29] a "case study" draws attention to what can be learned from a single case, and the goal of the study is to "...optimize understanding of the case rather then generalization beyond". While understanding the political process described here does not necessarily give valid grounds for empirical generalization, close study can give an interesting and valuable insight into the politics of local restructuring. This may again provide a basis for generating hypotheses in later research and may yield important contextual information. A single case study such as this is therefore best suited for answering "how" and "why" questions, not for rejecting hypotheses about causal relationships between different variables.

In gathering data from the Innlandet Hospital Trust, I followed the Trust closely over a period of three years. The challenge in this part of the data collection process was to maintain a close view in order to acquire a rich understanding of the processes but at the same time to keep the necessary distance from the actors involved in the process in order to avoid unwanted influence in either direction. The fact that the Trust partially financed the work that is presented here made keeping a distance between the researcher and the research subjects even more important. This has, however, not influenced any of the conclusions that are drawn in this article.

## Results

### Case study of a restructuring process

In 2002, Health East RHA was established as one of the five regional health authorities.^vii ^During the first year of service, Health East RHA established a local organization consisting of seven^viii ^hospital trusts with defined catchment areas. In addition, contracts with private service producers, including two private nonprofit hospitals (Diakonhjemmet and Lovisenberg), were established with their own defined catchment areas. One of the seven health trusts was Innlandet Hospital Trust (IHT), which had a catchment population of about 390,000 citizens and covered an area of 53,200 km^2^. IHT included hospitals from two former counties, Hedmark and Oppland, comprising six acute hospitals (county in parentheses): Gjøvik (Oppland), Lillehammer (Oppland), Kongsvinger (Hedmark), Elverum (Hedmark), Hamar (Hedmark), and Tynset (Hedmark). The largest proportion of the population was situated around Lake Mjøsa, which also divides the major cities and the main hospitals in the two counties. From 1985, a bridge across Mjøsa replaced the ferry and reduced travel time between the main cities (Lillehammer, Gjøvik, and Hamar) to approximately 30-35 minutes.

**Figure 3:**
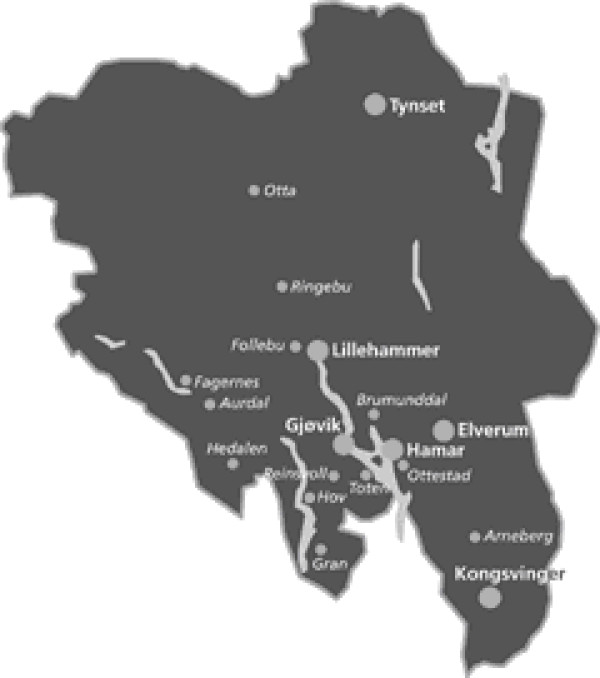
**Hospital units in the Innlandet Hospital Trust**.  Source: http://www.sykehuset-innlandet.no.

The main ambition of the IHT was to increase coordination between the largest hospitals, located in Lillehammer, Gjøvik, Hamar, and Elverum (see the map in Figure 3, which highlights the main hospitals with larger circles). As one of the informants in a study by S. Nerland [8] observes:

The motive was the situation around Lake Mjøsa. Attempts to coordinate across the county borders have been made for decades, but it was not possible under the old ownership. In 2002, we saw the outline of the same problem occurring between the hospitals.^ix ^Gathering all the hospitals in one trust was a deliberate organizational strategy.

Although not explicitly stated, it is believed that the idea of establishing one large hospital trust for the Lake Mjøsa region had support among key players in the Health East RHA that had its main office in Hamar, one of the cities in the catchment area for the IHT. For the maternity care services in the area, the Eastern RHA stated that "duplication of supply" was to be minimized, and "efficiency enhancing measures" were to be implemented. It was also stated "...decentralized units shall in principle be coordinated to strengthen the professional environments, take advantage of infrastructure, and increase the unit's robustness" [34]. I will structure the empirical description of the restructuring of health services in the IHT into two phases:

-- a first phase (2003), in which the main goal was to merge similar departments from two or three hospitals into a single department located at a specific hospital; and

-- a second phase (2004-2006), in which the main goal was to plan the future structure of the acute hospitals.

All of the data used in this presentation are qualitative. I followed the development of the trust over the period 2004-2006, and interviewed both the leadership group in the trust and one of the primary initiative takers behind the local protest movement in Gjøvik. In addition, I observed two meetings in the "samfunnspanel", and questioned both the former Minister of Health and an MP from the opposition about the process in interviews. Not all of the information gathered is included directly in the article, but it has served as important background information. In addition to these sources, local newspapers^x ^provide sources for quotes and information.

### The first phase: The vision of a functional organization

In the first phase, the IHT made plans to restructure hospital services grounded in a model of functional organization. The process leading to this proposal had been carried out without any involvement from local political actors, and the board meetings had been closed to the public. Although the proposal did not suggest closing down any acute hospitals, it was clear to all that the outcome of the process could lead to the centralization of healthcare services and longer travel distances, as not all hospitals in the future would supply all services.

The strongest reactions were observed in the city of Gjøvik. The maternity ward at Gjøvik hospital was to be closed and moved to Lillehammer where a new "women and children's clinic" was planned. A movement to save the maternity care unit in Gjøvik was soon established following initiatives by employees at Gjøvik hospital, local politicians, and trade union representatives. These actors then began to put their organizational and political affiliations to use. According to the founder of what was to become a protest movement to save the maternity ward in Gjøvik,^xi ^employees at Gjøvik hospital launched a petition before the final decision was made. Thus, the opposition against the trust started early. Moreover, even though local politicians no longer had any formal role in the decision-making process, they soon began making their voices heard at different venues, including an interpellation debate in the Gjøvik local council. The local political parties also put pressure on their representatives in parliament and the question quickly moved to the national political arena. In the parliament's question time on the 5 November 2003, a local MP confronted the Minister of Health with the restructuring plans and the minister signaled that he would overrule the decision made by IHT to move the maternity ward from Gjøvik to Lillehammer.

The Minister had probably made up his mind long before, and he had already organized an extraordinary assembly meeting for the Eastern Regional Health Authority on 6 November 2003. Here, he changed the previous decision. The maternity ward in Gjøvik was to remain untouched. In a press statement, the Ministry of Health acknowledged that the previous decision of the IHT was in accordance with national health political guidelines on the organization of maternity care, but arguments relating to the number of births in the existing ward were to be given higher priority [35,36]. It would appear that there was little or no opposition to this decision in parliament, as pointed out by one MP in an interview:^xii^

I do not think that there was a single politician in the Storting that thought this was right... The fear that this could become a pattern was there, at least for me. I hesitated a bit before saying that this should not happen, but still: This cannot take place all over the country! Then we would be making a mistake, and certainly I could not face my voters and others in my county and say that this was OK.

This particular MP clearly feared that this could become a pattern and he would not support a similar case if it took place in his county. This illustrates two important points. First, the Innlandet Hospital Trust was one of the first "tests" of the new organizational structure following the hospital reform. Other trusts/reformers may have learned from this process and reevaluated the potential for reform in their own area. Second, this serves as an additional reason for why no one opposed the protesters of Innlandet and the MPs from Oppland. The decision by the Minister of Health was in tune with the political preferences of the majority in parliament and local protesters and politicians.

In sum, this example from the first phase shows that the new board managed to reach a decision but was unable to implement it. The political protests were too loud to ignore, as both the Minister of Health and the district MPs quickly mobilized, and the Minister of Health decided to overrule the decision made in the IHT. Clearly, IHT did not have the degree of power over the issues that the formal structure suggests. As a response to the intervention of the Minister of Health, the chairperson of the IHT board (a local business leader) decided to resign. In a local newspaper, he commented on the Minister's intervention as follows:

He is in his full right to overrule the board's decisions, but this means that we are on our way back to the old political model of governing [37].,

### The second phase: The 2020 project

Health East RHA now appointed the deputy director of the Health East RHA as the new chairperson of the IHT board. Despite the turbulence experienced in the first phase following the creation of the IHT, the trust's leadership set out to develop a long-term investment plan known as Project 2020 [38]. Having learned from Phase 1, IHT chose to organize the process in this phase differently. First, the process was to be open and inclusive. Second, the trust invited politicians from the municipalities and the counties to take part in a new political advisory group. The process in Phase 1 showed the trust that actors in its close environment had a considerable degree of control over events that were crucial to them. The CEO of IHT described the trust's experience thus far when he addressed the new advisory group:

When specific cases reach the agenda the uproar starts. We face three types of actors: the media - our own employees - and the politicians. These three form alliances that are more powerful then the trust's own alliances with each of them. How can we form equally strong alliances? [39],

The mayors of two of the municipalities, Hamar and Stange, replied that forming this new advisory group could be a wise first step towards achieving an alliance [39].

One of the most important questions for the project was the establishment of the main hospital in the region. Signals soon pointed to Hedmark as the county where the main hospital would be sited. This made the conflict escalate once more and the trust faced criticism from a wide array of actors, including politicians from all levels of government, employees, and other actors who had joined the protests. The conflict escalated even further when the CEO made it clear that it would be difficult to maintain current emergency capacity at all the units within the trust. A physician at the unit in Lillehammer (in the county of Oppland) illustrates how the trust's actions were interpreted:

Imagine that your neighbor, for all to see and without anyone intervening, robs and steals the most valuable items in your house. He does this easily and calmly because it is perceived as "legal", and places them in his own house. A few months later, he has the nerve and indecency to invite you in to see how rational and beautiful his house has become. At the same time, he criticizes your house for its misery. THIS is what has happened [40].,

This shows how emotional the conflict was and that the trust's own employees shared the views of groups in the organization's surroundings. Developments after the ownership reform were seen as a process where the leadership of the newly formed hospital trust deliberately wanted to take from Oppland and give to Hedmark. This was a formidable challenge for the trust. In one of the meetings of the new advisory group, the CEO described the situation in the following way:

We have heard the accusations that we want to put everything in Hamar, we have even been called "Hamar mobsters". Therefore, we have a need to be seen as credible, to practice openness .... [41],

The relationship between the trust and the political actors in its surrounding area was close to a warlike situation at that time, with the CEO acknowledging that it was not possible to administer the trust in such a situation. He attempted to solve this problem by giving local and regional politicians more influence in the decision-making process [41]. However, this did not work and protests escalated even further. For example, all of the MPs from Oppland [42] wanted to divide IHT into two organizations, i.e., they wanted to return to the situation that preceded the formation of the IHT. Almost simultaneously, the chief physicians of the hospitals located in Oppland county signed a petition demanding resignation of IHT's leadership. Even the central state's local representatives, the county governors,^xiii ^joined in. The state prefect in Hedmark argued in a statement that:

It is necessary to gather the most specialized functions so the patients in Innlandet can receive specialist care of the same quality as patients elsewhere in the country. What is best for the patients must come before political considerations [43].

The county governor from Oppland County viewed the situation somewhat differently:

... a health care service that used to be very good is now being dramatically reduced, and as many view it, to the benefit of Hedmark. Important functions are now being moved out of the county [43].

This phase also ended with a backlash against IHT. What had started as a plan to discuss the hospital structure of the whole region ended only with a discussion of a new hospital structure for the Hamar-Elverum area. The CEO of IHT summed up the plans in a newspaper interview in May 2006:

The hospitals in Elverum and Hamar (read: in Hedmark) will become "twins" and function as a central hospital for Hedmark. We no longer presuppose that patients will need to cross the county boundaries in order to receive care [44].

When asked by the journalist whether this meant they were back where they started from several years ago, the CEO replied:

It may seem that way for some, but I think the end result of this process is an acceptable result both for Hedmark and Oppland. We have a long way to go before we can have one hospital for the entire region, where all functions are gathered in one hospital. It might also be the case that we will never see that happen [44].

## Discussion

The question posed in this article was whether the 2002 hospital reform facilitated the restructuring of the hospital sector. The case of the Innlandet Hospital Trust stands out because it was the first large scale attempt to change structures after the change in ownership in 2002. Based on the findings of the IHT case, it appears that the ownership reform did not facilitate hospital restructuring. Whether this reflects a more general pattern is an empirical question. A single case study does not create a basis for empirical generalization and we cannot, based on the findings in this case study, conclude that the same factors are at work in other reform processes. What is clear, however, is that similar processes took place in other areas and that local protest groups played key roles in other restructuring processes [45-47]. Networks of politicians and others were created, and a formal national network was formed, with a focus on influencing national politicians and stopping changes in local hospital services that involved changes in emergency capacity [48]. Based on the findings, two factors explain that restructuring of the hospital sector may be as difficult as it was before the reform.

First, the actual policy process regarding local reforms is far more complex than could be predicted by simply focusing on the formal delegation structure of the new organizational model. During the first phase, the IHT learned that local actors were influential and, as a direct consequence, they reintroduced politics into the process by creating a new arena for this purpose. The rationale for this was clearly to trade influence in the decision-making process against support, but this was insufficient for the local actors. Based on the comments made by the leadership of the trust, this was a forthright attempt to trade influence against support. Another interpretation is that the trust attempted cooptation [49]. Regardless of which interpretation is correct, the attempt shows that the trust acknowledged that they could not ignore local politicians. Nevertheless, in order to be effective, this had to be attractive for local politicians, and this was not necessarily the case. An interesting comment about the role and incentives of local politicians was made by the chief editor of one of the largest newspapers in the region following one of the meetings of the new advisory committee for the IHT:

They (the politicians) don't have to assume responsibility, and they will blame you (the trust) no matter what [39].

Thus, the trade-off of support vs. influence was never attractive for local politicians. They never accepted the trust's decisions regarding hospital structure, despite increased involvement in the decision-making process. In any case, the process described here shows that politicians were not made redundant when the central state took over ownership. Local politicians and other protesters created an environment for the trust that made restructuring even more difficult and could function as an important link to politicians at the national level. Clearly, the trust did not have the de facto power to reform the hospital structure in the way they saw fit, despite having the de jure power to do so. This observation is almost trivial, as studies of policy implementation [50,51] have provided similar insights a number of times before. The growing literature on governance [52-54] points in the same direction and is premised on an understanding of the inability of central states to govern. The central state has to take into account the preferences and aims of multiple actors. In the IHT case, local protest groups were formed and local and regional politicians played an important role. Members of parliament from the same district face the same incentives as they are elected on the basis of votes in their county of nomination. Standing firm in order to secure a possible long-term benefit by improving the cost control and efficiency of the hospital sector is probably of less value for their local voters than engaging in, and supporting, local protests. Thus attempting to "remove politics" in order to increase decision making capacity may be futile, significant local restructurings of the hospital sector are inherently political issues.

The second factor relates to the vision of the national goals of an efficient hospital sector spelled out in the report from the royal commission that considered the central government takeover of the hospitals [4]. How clear is this vision, if at all? As discussed earlier, a problem under county ownership was that the county politicians had to balance local political considerations against the national goals of an efficient hospital structure [4]. However, national politicians also need to balance these goals, and there is no ex ante reason why their preferences should differ from local and regional politicians. As shown by Nerland [8], politicians at the national level have actively reintroduced politics to many local reform processes, and the involvement of MPs in local hospital issues is far higher than predicted from the intentions of the reform [55]. In the case of the maternity wards, the ambiguity in the signals sent to the RHA becomes clear by taking a closer look at the statements made during the 2003 budgets process. At the general level, the statements are in favor of reform:

The majority in the committee, being members from Labour, the Conservatives, the Progress party and the Christian Democrats, means that there can be a need for changes also in the local hospitals. Professional deliberations may lead to necessary changes in maternity care and emergency capacity. It is not politically possible to appropriate oneself out of this need for reforms. Parliament will then have to rely itself on the advice given by important professional communities such as the national board for maternity care [56].

In 2007, the members of the national advisory board for maternity care, established to provide professional guidance and advice in reforms relating to maternity wards, left their posts in protest.^xiv ^The leader of this group, Professor Pål Øian at the maternity clinic in Tromsø, is quoted as saying that:

The RHA started out with a clear mandate and a unanimous resolution from Parliament, but faced massive resistance from local politicians, local media and local professionals, and they received no signals from the Ministry of Health and Care that they would receive any support when implementing these resolutions [57].

How clear the formal mandate given to the RHA actually was is unclear. Formally, they could organize healthcare services in the way they wanted, and were clearly expected to do so in order to reduce costs [58], but just how far these reforms could go was unclear. The Innlandet Hospital Trust represents one of the first major tests in this respect. Similar challenges are also present in other areas/trusts. The director of another trust, the Blefjell Hospital Trust, argues that the trusts face a dilemma:

The demand for economic balance combined with unwillingness to accept significant structural changes, may lead to decreased quality. Either you have to fund the existing hospital structure, or you have to accept reforms [59].

In the case of the maternity ward at Gjøvik, the National Board for Maternity Care had declared that the creation of one maternity clinic in Lillehammer (which involved shutting down the department in Gjøvik) was justified on medical criteria. The total quality of the services available for the population in Gjøvik would not necessarily decrease if this proposal were implemented [35]. However, ultimately the Minister of Health decided that the centralization of the maternity ward was not only a medical and economic question but also one of geography and redistribution, and therefore politics. As the minister himself replied to a MP who wanted all decisions that involved shutdowns to be handled by Parliament:

Whether or not something is of principal importance will of course have to depend upon political considerations (Answer to Olav Gunnar Ballo 24.01.2002, see also [8], page 155)

The Minister of Health could rationalize his statement by referring to the Hospital Trust Act of 2001 [7], where it was declared that matters that were of a principal importance were to be presented to the Minster of Health. It is, of course, difficult for local decision makers to know *ex ante *what constitutes a matter of principal importance. However, the combination of early intervention in the case of Gjøvik and the *ex post *potential for the involvement of politicians at all levels of government has lead to ambiguity. The central state encouraged local restructuring in order to reduce costs and thereby budget deficits in the sector [58], but it did not accept the consequences.

## Conclusion

Reforms such as the one that is described in this article are inherently political. Voters pay attention to any changes in the local hospital structure that may have (perceived or real) negative effects on the services that are provided in their area. Keeping politics at an arm's length may simply be unrealistic and further complicate the politics of local hospital reforms. However, it is important to emphasize that this study does not provide grounds for empirical generalization. We cannot, based on this study alone know just how particular the process that is described and analyzed here is. Whether the same type of mechanisms that have been described in this article are relevant in similar processes in other countries remains an open empirical question. This case study does however show that an attempt to keep politics at arm's length in questions such as these may be futile.

The Norwegian hospital reform of 2002 was an attempt to simplify the organizational structure of the hospital sector. By gathering responsibility for all issues into one hand, the central state, the general governing of the sector was expected to improve. This case study indicates that the reform paradoxically may have made the process more complex. The combination of political interaction, both on the vertical plane between political actors at different levels and on the horizontal plane between political actors and other actors outside the formal political arena, created a highly complex political environment for the hospital trusts. Local networks with politicians as their key figures, in combination with employees and other actors, demonstrated that they had more power over local developments in the hospital structure than the formal organizational structure of the healthcare system suggested. At the same time, politicians at the central level of government were vulnerable to local protests and did not appear to accept the consequences of their demand for balanced budgets in the RHA.

## Competing interests

The author declares that they have no competing interests.

## Appendix

i. This means that the Ministery of Health is not expected to play an active role in steering the RHAs. The central states channel for direct influence is in the enterprise meeting, this enterprise meeting can be compared to a general assembly in a limited company.

ii. The Stoltenberg I cabinet came into power on the 22 March 2000. However, at this time there were few or no signals of the large reform in the hospital sector that were to follow shortly after, although Stoltenberg stated that he wanted to "renew" the public sector. A more detailed description of the process behind the Norwegian hospital reform can be found in the works of Nerland [8], Berg [9] and Hagen and Kaarboe [10]

iii. Tjerbo and Hagen [11] provide a more elaborate discussion of these deficits and what caused them and is also the source for figure 1.

iv. By visible, I mean policies that voters perceive as being important. First, any negative effects of policies in this area have the potential to be a matter of life and death. Second, all voters know that they or someone near them are likely to be in need of these services sometime in the future. This is also an explanation of why local reforms that alter traveling time and availability are controversial and why equality of access is the key health political goal in Norway. This is problematic in that the effects of any proposed change on equality of access are not always straightforward and the positive effects of other variables may outweigh the negative effects. This is illustrated by the proposed change in maternity services shown later in the study.

v. In the bill that switched ownership, matters of "principal importance" are to be presented to the Minister of Health. This means that extraordinary general assemblies can be held when matters of a principal nature reach the agenda. Just what exactly constitutes a matter of principal importance, however, is not stated. Confer §30 in Ot.prp. nr 66 (2000-2001).

vi. Some do however claim that this is a misunderstanding and that generalization can be achieved also in case studies [29]. For a more in depth discussion of the case study method see Yin [28], Stake [29], Flyvbjerg [30], Gerring [31,32] and Lijphardt [33].

vii. The number of RHAs fell to four from 1 July 2007 with the amalgamation of Health East RHA and Health South RHA into Health South-East RHA.

viii. The correct number is eight if Sykehusapotekene ANS (Hospital pharmacies ANS) is included.

ix. In 2002 (the first year after the reform), the hospitals were preliminarily organized along county lines.

x. It is important to keep in mind that these local newspapers can be seen as active participants in the process in their own right. A somewhat similar argument applies regarding the use of the internal information paper *InnSi'a *(see reference no. 39). However, I do not see that this has in any way lead to a biased depiction of the process that is described here. I rely on several sources of information and these local newspapers are primarily used as a source of quotes from central actors.

xi. Personal interview with Finn Olav Rolijordet on 7 October 2004.

xii. This interview took place on 2 June 2005. The MP represents one of the largest opposition parties and was not elected from Hedmark or Oppland.

xiii. The county governor is the central state representative in the counties. He/she functions as a regulative authority for the municipalities and as an organ to facilitate implementation of central state policies.

xiv. This protest was aimed at Sylvia Brustad, who was Minister of Health at the time. The members of the National Board for Maternity Care argued that the Ministry of Health and Social Care had ignored their advice and hindered reorganization of the maternity care services.

## Pre-publication history

The pre-publication history for this paper can be accessed here:

http://www.biomedcentral.com/1472-6963/9/212/prepub
